# Bioprospecting of serratiopeptidase-producing bacteria from different sources

**DOI:** 10.3389/fmicb.2024.1382816

**Published:** 2024-05-09

**Authors:** Sreelakshmi R. Nair, C. Subathra Devi

**Affiliations:** Department of Biotechnology, School of Bio Sciences and Technology, Vellore Institute of Technology, Vellore, Tamil Nadu, India

**Keywords:** *Serratia marcescens*, serratiopeptidase, anti-inflammatory drugs, fibrinolytic protease, SDS-PAGE

## Abstract

Anti-inflammatory enzymes have wide applications in the pharmaceutical industry. The objective of this study was to find new and efficient strains for the commercial production of serratiopeptidase enzyme. Vast number of samples were processed for the isolation of potent strains. The experimental treatment includes processing of twenty soil samples, silkworm gut, and sugarcane stem. The total protein and protease activity was estimated by Lowry’s method and casein hydrolysis. The HRBC stabilization assay was performed for finding the anti-inflammatory potential of all strains. The serratiopeptidase production was confirmed by HPLC with the standard. Molecular characterization of selected potent strains was done by 16S rDNA and confirmed the taxonomy. The one step rapid purification of serratiopeptidase was performed by Ultra three phase partitioning method. The clot lysis potential of the *Serratia marcescens* VS56 was observed by modified Holmstorm method. The results of the study revealed that among the 60 strains, 12 strains were protease-positive on skim milk agar plates and showed significant protease activity. All 12 strains were screened for serratiopeptidase using high-performance liquid chromatography (HPLC) and VS56, VS10, VS12 and VS18 showed a similar retention time (4.66 ± 0.10 min) with standard. The selected potent strain, *Serratia marcescens* VS56 showed a proteolytic activity of 21.30 units/mL and produced a total protein of 102 mg/mL. The HRBC suspension results also showed a percentage of 94.6 ± 1.00 protection, which was compared to the standard diclofenac. The clot lysis potential of *Serratia marcescens* VS56 was 53% in 4 h. Furthermore, the molecular weight of the protein was identified to confirm the presence of serratiopeptidase. The study hence contributed successfully to isolating, screening, and identifying a potent producer for serratiopeptidase from an environmental source. This inherent advantage of the strain will undoubtedly contribute much to the coco comm commercial production of serratiopeptidase in the near future.

## 1 Introduction

Serratiopeptidase is a serine protease enzyme with potent anti-inflammatory, wound healing, and anti-biofilm properties (Tasaka et al., 1980). This enzyme has a molecular weight of 60 kDa and is widely used in tablets and injection suspensions (Devi et al., 2013; Nair, 2022). Serratiopeptidase has a crucial affinity to enzymes such as cyclooxygenase I and II. It also influences the production of interleukins, prostaglandins, and thromboxane, which are released in the arachidonic pathway (Steiger and Harper, 2014; Khosravi et al., 2023). Serratiopeptidase can hydrolyze the molecules such as serotonin, histamine, and bradykinin, which can cause swelling (Abbas et al., 2009; Navneet Kumar et al., 2017). The enzyme is available in various forms, such as tablets, injection suspensions, and combination drugs. The bacterial production of serratiopeptidase was once a widely used industrial method. Currently, the enzyme industry primarily relies on bacterial enzymes such as pectinase, hydrolases, and transferases (Gupta et al., 2015). Serratiopeptidase was considered one of the most needed enzymes in various industrial sectors. Our study represents one of the most elegant approaches in the identification of serratiopeptidase-producing strains from various sources. Serratiopeptidase and its rising demand in pharmaceutical industries peaked during the COVID-19 pandemic. Since bradykinin, histamine, and serotonin are hydrolyzed by serratiopeptidase in analgesic reactions (Desser et al., 1993; Ferrero-Miliani et al., 2007), this proteolytic enzyme is known to reduce post-COVID-19 symptoms such as nasal congestion and edema (Bhagat et al., 2013; Sharma et al., 2021). The application of serratiopeptidase is emerging in the medical and pharmaceutical sectors, where it is an essential ingredient in many drug formulations. Therefore, there is an urgent need for potent strains for the large-scale production of this enzyme. The native producer of serratiopeptidase is a known pathogen and has been isolated from infectious sites. This limitation complicates the industrial handling of this microbe (Pansuriya and Singhal, 2010). Furthermore, any industrial strain, when used for a longer period, tends to lose its yield and become a minimal producer. This study focuses on one such wild strain with effective serratiopeptidase production. The current research is therefore intended to isolate, screen, and identify a potent serratiopeptidase-producing bacterium from various sources. Soil isolate can be considered a safer source for industrial and commercial production.

## 2 Materials and methods

### 2.1 Sample collection

Different types of soil samples, such as slaughterhouse soil, agricultural waste soil, fish market soil, rhizosphere soil of tomato, ladies finger and sugarcane, mangrove soil, coconut waste, rhizosphere soil from mulberry plant, silkworm gut, and sugarcane stem, were collected from different areas of Tamil Nadu and Kerala. Different soil samples were collected 5–10 cm below the surface (Das and Prasad, 2010; Rebecca et al., 2013).

### 2.2 Isolation and screening

A measure of 1 g of soil was serially diluted in physiological saline (0.85%) and spread plated onto nutrient agar plates (0.5% peptone, 0.3% beef extract/yeast extract, 0.5% NaCl, 0.5% agar, and pH 7 ± 0.4) and incubated at 37°C for 24 h. Silkworm was dissected aseptically to obtain the gut and was mixed with 0.85% saline, which was plated onto a nutrient agar medium (Zhang et al., 2020). The sugarcane stem was surface sterilized using 70% ethanol and used for isolation on a nutrient agar medium. After the incubation, the colonies were selected and further screened for protease activity (Viswanathan et al., 2003).

### 2.3 Morphological and biochemical characterization of protease-positive strains

The selected strains were identified by morphological and biochemical characterization. The 16 h old culture of VS02, VS03, VS05, VS10, VS11, VS12, VS15, VS16, VS18, VS25, VS44, and VS56 was used for biochemical analysis. The results were analyzed using Bergey’s manual of bacteriology (Bergey, 1994; Caprette, 2009).

### 2.4 Molecular characterization of selected strains

All five selected strains were subjected to 16S rDNA sequencing. DNA was separated from the strains and 5 μL of the test was added to 25 μL of PCR mixture, which contained 5 μL of deionized water, 1.5 μL of forward primer (5’-AGAGTTTGATCTGGCTCAG-3′), and 12 μL of Taq Expert Blend and reverse primer (5’-TACGGTACCTTGTTACGACTT-3′). The enhanced 16S rDNA arrangements were submitted to NCBI (Altschul et al., 1990). The FASTA was assessed using the program MUSCLE 3.7 (Edgar, 2004). MEGA 11 software with bootstrap values was utilized for the examination of phylogeny (Kumar et al., 2016). The 16S rDNA sequences were submitted to GenBank, and accession numbers were generated.

### 2.5 Total protein estimation using the Lowry method

The total protein produced by selected strains was estimated by the standard method of Lowry and Folin, using bovine serum albumin (BSA) as the standard. The cell-free supernatant was taken as the sample for the analysis (Lowry et al., 1951).

### 2.6 Protease activity

The isolated strains were examined for protease activity on skim milk agar plates (10% skim milk powder, 0.5% peptone, 0.5% NaCl, and 1% agar) (Jo et al., 2008). The primary screening was performed with 10% skim milk powder (Araghi et al., 2019). After a 24 h incubation at 37°C, the plates were checked for the zone of hydrolysis (Masi et al., 2021). The secondary screening for protease was conducted by cutting wells of 1 mm diameter on skim milk agar plates and adding 100 μL of cell-free suspension to the wells. The plates were incubated upright under 37°C 24 h. The zone of clearance was measured after incubation (Alnahdi, 2012).

### 2.7 Protease assay

Protease-positive strains were further analyzed for protease activity. A measure of 0.2 mL of supernatant was added to 2.0% casein solution prepared in Tris–HCl of pH 8.0 and incubated at 37°C for 10 min. The reaction was terminated by the addition of 1 mL of 0.1 M 10% trichloroacetic acid (TCA). This was further incubated for 15 min at 37°C. The reaction mixture was centrifuged at 10,000 rpm for 10 min. The supernatant was collected and added to 2.5 mL of Na_2_CO_3_ (0.44 M) and 1 mL of Folin–Ciocalteu reagent (1: 3 ratio). This was incubated in the dark for 30 min, and absorbance was calculated at 660 nm. The experiment was performed in triplicates. Protease production was observed using casein as a substrate (Cupp-Enyard, 2008; Saxena and Singh, 2011; Bhargavi and Prakasham, 2012).

### 2.8 Production of serratiopeptidase

The protease-positive strains were inoculated into 100 mL of tryptic soy broth (casein peptone 17 g/L, soy peptone 3 g/L, NaCl 5 g/L, dipotassium hydrogen phosphate 2.5 g/L, glucose 2.5 g/L, and pH – 7.3 ± 0.2) and kept for incubation at 37°C 24 h at 240 rpm and checked for protease activity. The total protein content and anti-inflammatory activity of protease-positive strains were estimated (Pansuriya and Singhal, 2010; Petersen and Tisa., 2014).

### 2.9 HPLC analysis

The screened protease-positive samples were further analyzed to confirm the presence of serratiopeptidase by performing HPLC. The standard serratiopeptidase was procured from Sigma Aldrich. The standard was prepared by dissolving 1 mg/mL in HPLC grade methanol and filtered through a 0.45 μm nylon filter. The retention time of the standard was compared to the samples (Navneet Kumar et al., 2017; Pushparaj, 2017). The mobile phase used was methanol and water in a ratio of 50:50. HPLC grade solvents were filtered through a 0.45-μm nylon filter.

### 2.10 Anti-inflammatory activity

All protease-positive strains were taken for the anti-inflammatory analysis. Blood was collected from the blood bank, and an equal volume of Alsever solution (2% dextrose, 0.8% sodium citrate, 0.5% citric acid, and 0.42% sodium chloride) was added. The solution was centrifuged at 3000 rpm, the packed cells were washed with iso-saline (0.9% w/v NaCl) and then a 10% solution was prepared. A measure of 1 mL of supernatant, 1 mL of phosphate buffer, 2 mL of hyposaline, and 0.5 mL of human red blood cell (HRBC) suspension were added. This was centrifuged for 20 min at 3000 rpm and incubated for 30 min at 37°C. The hemoglobin content of the supernatant solution was estimated spectrophotometrically at 560 nm. The experiment was conducted in triplicates. A measure of 1 mg/mL of diclofenac was used as standard, and the control was without the supernatant (Joseph et al., 2013). The percentage of protection was calculated using the formula. The percentage of protection was compared with standard (diclofenac).

Percentage of protection = 100−(Abs of sample−Abs of control/Abs of control x 100).

### 2.11 Clot lysis activity

Clot lysis was conducted using the modified Holmstorm method (Holmström, 1965). A measure of 500 μL blood was added to each sterile, pre-weighed separate tubes. All the tubes were incubated at 37°C for 45 min, and the plasma was carefully removed after clot formation. To all tubes, 100 μL, 200 μL, 300 μL, 400 μL, and 500 μL of crude enzyme of *S. marcescens* VS56 were added. This was incubated for 2–8 h at 37°C. Following incubation, the lysed clot was removed, and the weight of the remaining clot was calculated. After every 2 h of incubation, the weight of the clot was calculated. This was repeated as singlets, and the percentage of clot lysis was found using the formula.

Percentage of clot lysis = (weight of lysed clot/weight of clot x 100).

### 2.12 Purification of enzyme

Ultra three-phase partitioning (UTPP) was performed with slight modification. The experiment was carried out using 30% (w/v) saturated ammonium sulfate (Salarizadeh et al., 2014; Chander et al., 2021) with a pH 7.0 and 1:1 (v/v) ratio of t-butanol to crude. The mixture was kept overnight at 240 rpm, after which the precipitate was separated out. It was then dissolved in 50 Mm sodium phosphate buffer (pH 7 ± 0.2) and ultrasonicated at a frequency of 20 kHz for an irradiation time of 5 min (Pakhale and Bhagwat, 2016). Finally, the sonicated samples were dissolved in phosphate buffer (pH 7 ± 0.10) and stored.

### 2.13 SDS-page

The identification of protein produced by *S. marcescens* VS56 was conducted using SDS-PAGE (Salamone and Wodzinski, 1997). The crude and completely purified samples of *S. marcescens* VS56 were run in sodium dodecyl sulfate-polyacrylamide gel electrophoresis (SDS-PAGE), with 4% stacking gel and 12% separating gel. Both purified and crude samples were used for SDS-PAGE analysis. The protein marker in the medium range was used to compare the protein size obtained (Devi et al., 2013).

## 3 Results

### 3.1 Isolation and screening

**Figure 1:**
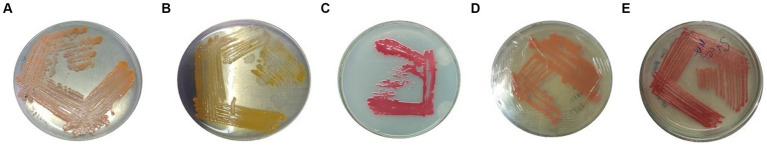
Pure culture of **(A)** VS10, **(B)** VS03, **(C)** VS12, **(D)** VS15, and **(E)** VS56 on the nutrient agar medium.

**Figure 2:**
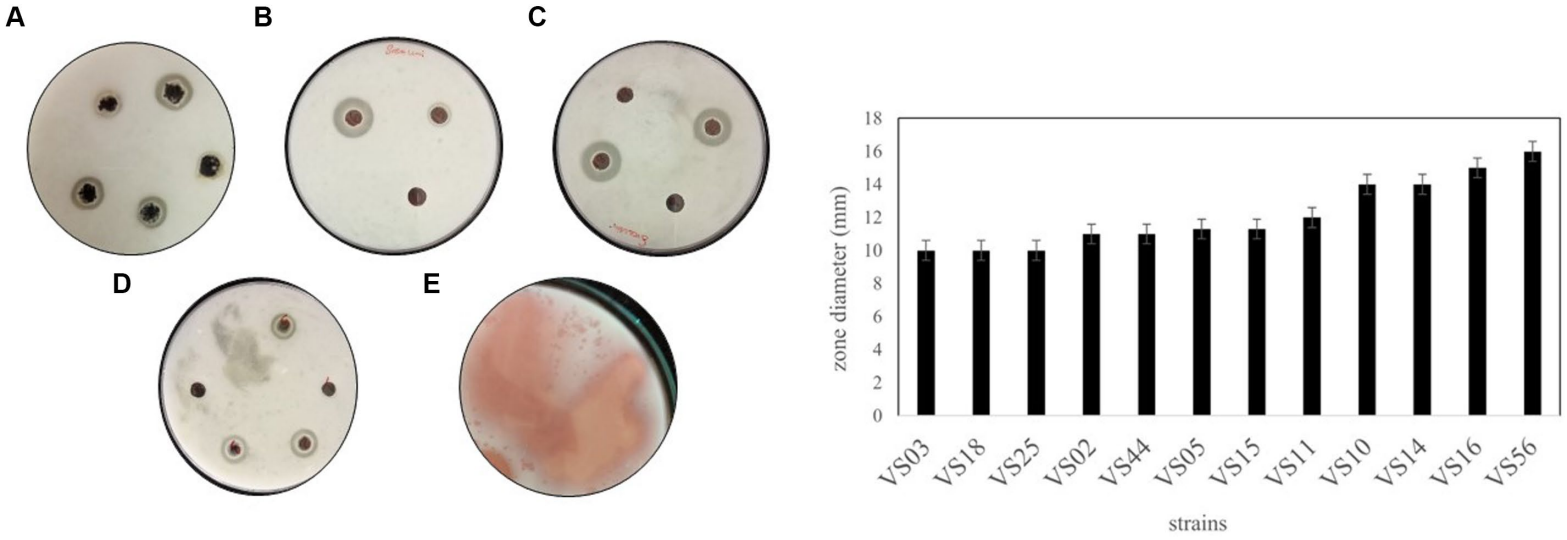
A-1 Zone of hydrolysis on skim milk agar **(A)** VS02, VS03, VS05. **(B)** VS12, VS44. **(C)** VS16, VS10. **(D)** VS18, VS24, VS44. **(E)** VS56. **(B)** Zone diameter of VS02, VS03, VS05, VS10, VS11, VS12, VS15, VS16, VS18, VS25, VS44, and VS56.

Among all the processed samples, 60 strains were isolated from different sources. The pigmented and non-pigmented colonies were selected, and pure cultures were maintained for further examination on nutrient agar plates (Figure 1; Supplementary Figure 1). The strains were named VS1-VS60 and were further screened for protease activity in skim milk agar medium. The total protein and protease activity of all strains were observed. From 60 isolates,12 strains exhibited a zone of hydrolysis on skim milk agar. This showed an indication of protease production. Upon streaking onto skim milk agar (10% skim milk powder), the zone of hydrolysis was observed around the streak lines (Figure 2). Furthermore, secondary screening of the strains VS02, VS03, VS05, VS10, VS11, VS12, VS15, VS16, VS18, VS25, VS44, and VS56 showed good zone of hydrolysis. Among all, VS56 has a maximum zone diameter of 16 mm and excellent protease activity (Figure 2). The strain VS56 has excellent casein degradation potential. When compared to other strains VS10, VS16, and VS12 have moderate hydrolytic activity, and VS03, VS18, VS25, VS02, VS05, and VS44 have good hydrolytic activity (10 mm). Based on primary and secondary screening, VS56 was identified as a potent protease producer.

### 3.2 Morphological, biochemical, and molecular characterization of potent strains

**Figure 3:**
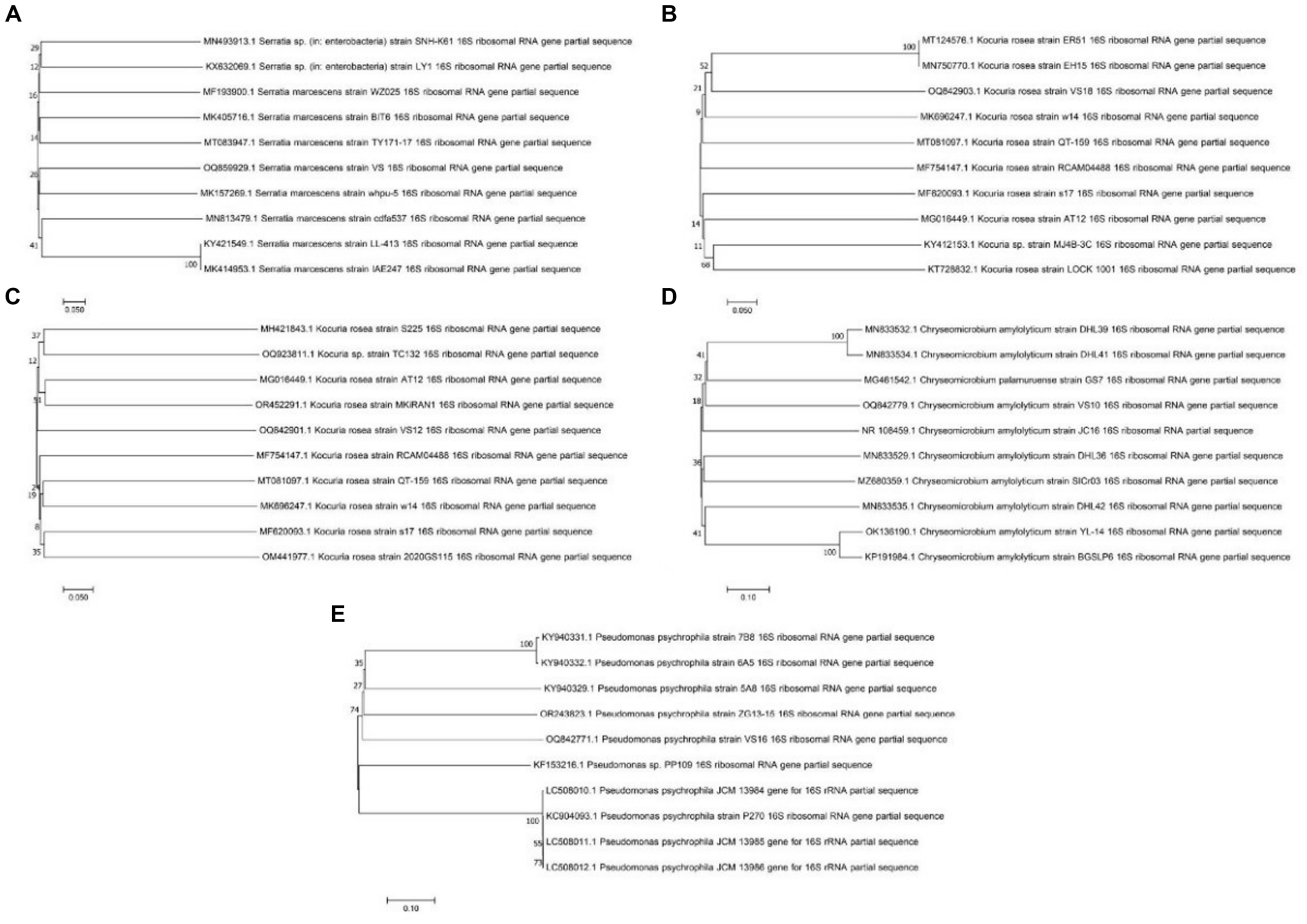
Phylogenetic tree of **(A)** *Serratia marcescens* VS56, **(B)** *Kocuria rosea* VS18, **(C)** *Kocuria rosea* VS12, **(D)** *Chryseomicrobium amylolyticum* VS10, and **(E)** *Pseudomonas psychrophila* VS16.

Morphological and biochemical characteristics of strains having maximum protein and anti-inflammatory activity were identified at the species level (Table 1). The selected strains had similar retention times in the HPLC chromatogram when compared with the standard. The species-level identification revealed that the potent strains were *S. marcescens* VS56 (OQ859929), *Chryseomicrobium amylolyticum* VS10 (OQ842779), *Kocuria rosea* VS18 (OQ842903), *Pseudomonas psychrophile* VS16 (OQ842771), and VS12 *Kocuria rosea* (OQ842901; Figure 3).

**Table 1:** Morphological and biochemical characteristics of selected strains.

Strains	Colony morphology on nutrient agar	Gram staining	Indole	MR	VP	Citrate	Nitrate	Catalase	Oxidase	Protease activity
VS02	Small round smooth colorless colonies with entire margins and raised surface	+ve	−	−	+	−	+	+	−	+
VS03	small golden yellow colonies with round smooth convex colonies, opaque smooth-edged.	-ve	−	−	+	+	+	+	−	+
VS05	Smooth, circular, white colonies, irregular, flat colorless colonies.	-ve	−	−	−	+	−	+	+	+
VS10	Red-colored large round rough-edged colonies.	+ve	−	−	−	−	+	+	+	++
VS11	Large creamy white, circular, raised sticky colonies.	-ve	+	+	−	−	+	+	−	+
VS12	Red-pigmented circular, slightly convex, and smooth surface colonies.	+ve	−	−	−	+	+	+	+	+++
VS15	Red pigment-producing colonies with a circular, slightly convex, and smooth surface	+ve	+	−	−	+	+	+	+	+
VS16	Yellow-colored large, opaque, flat colonies with irregular margins and a pleasant odor.	-ve	−	−	−	+	+	+	+	++
VS18	Large, orange-colored opaque flat colonies, slightly convex, and smooth surface.	+ve	−	−	−	+	+	+	+	++
VS25	Red pigment-producing colonies with circular, slightly convex, and smooth surfaces.	-ve	+	−	−	+	−	+	+	+
VS44	Orange-colored, large, smooth, entire bargained, convex circular colonies.	-ve	+	+	+	−	+	−	+	+
VS56	Large, concave, mucoid, circular red colonies	-ve	+	−	−	+	−	−	+	+++

+minimum activity, ++ moderate activity, +++ maximum activity.

### 3.3 Protease activity and total protein

**Figure 4:**
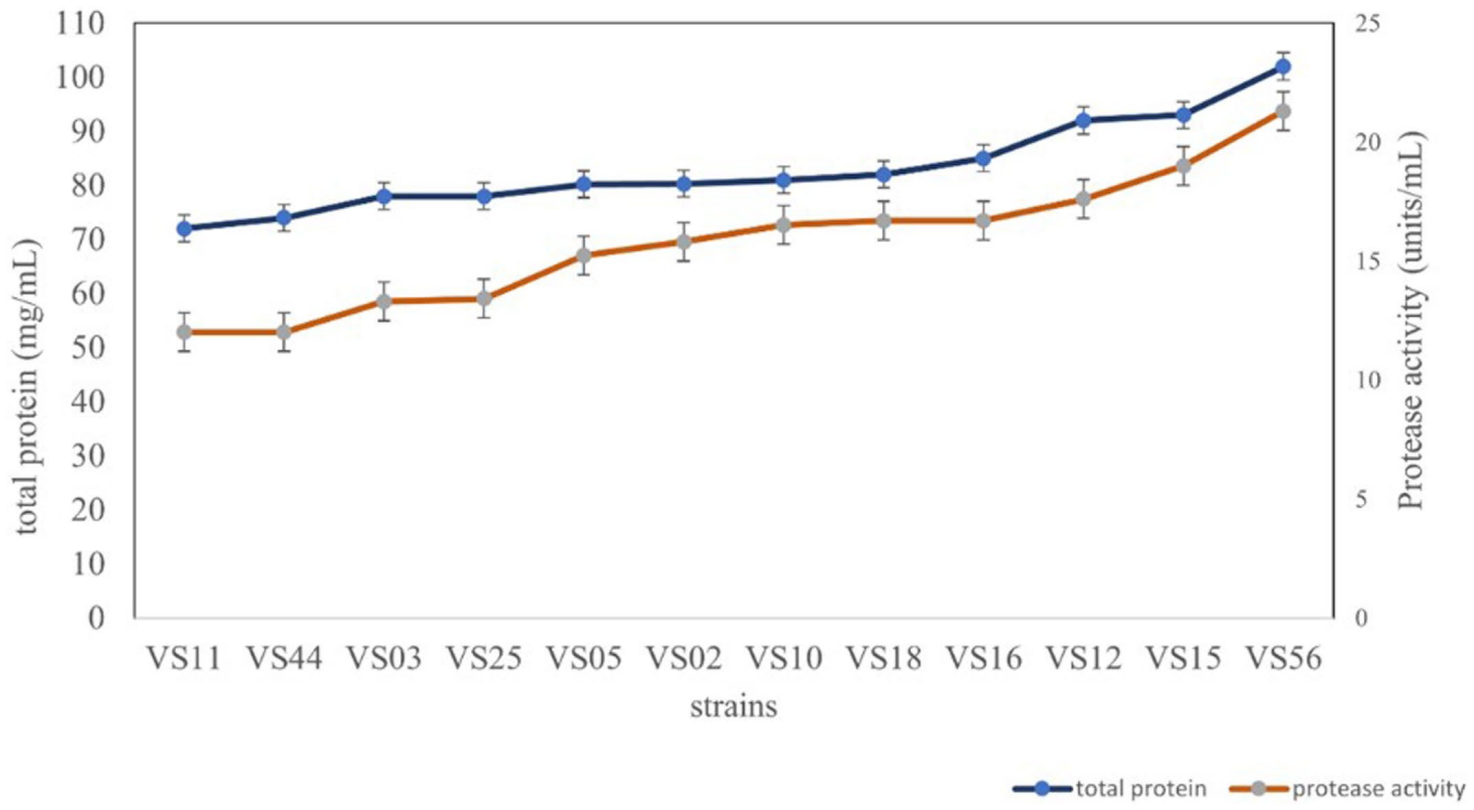
Protease activity and total protein content of strains.

All the selected strains were further analyzed for total protein and protease activity. Secretory proteins in all cell-free supernatants were estimated to be potent producers. The strain isolated from waste soil, VS56, showed a maximum protease activity and total protein content of 21.30 units/mL and 102 mg/mL, respectively (Figure 4). Proteolytic bacteria are widespread and have diverse sources for isolation. The sugarcane soil isolate VS18 and mangrove soil isolate VS11 and VS44 had the least protein content and protease activity (72 mg/mL and 12.31 units/mL). Strains isolated from agricultural land (VS03) and butcher soil (VS25) have 78 mg/mL of total protein and 13.12 units/mL of protease activity. Fish market soil serves as a rich source for proteolytic bacteria. Fish market soil isolate VS05, sugarcane soil isolate VS02, and silkworm gut isolate VS10 showed a moderate protein content of 80.20 mg/mL, 80.3 mg/mL, and 81 mg/mL, respectively. Furthermore, the protease activity of these strains was identified to be 15.32 units/mL, 14.84 units/mL, and 16.81 units/mL. The strains VS16 (tomato soil) and VS12 and VS15 (coconut waste) are potent protease producers with higher amounts of secretory protein. The sugarcane sample isolate VS11 has the least amount of protein (72 mg/mL) and protease activity (12.31 units/mL). The waste soil isolate VS56 has the most protease activity and active protein in it.

### 3.4 HPLC analysis

**Figure 5:**
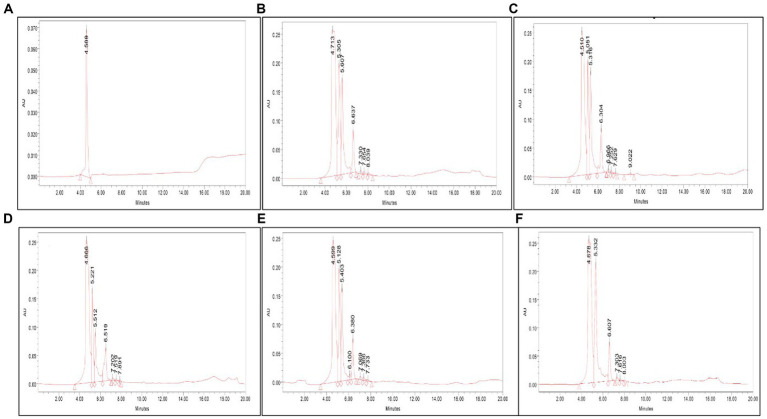
**(A)** HPLC chromatogram of **(A)** standard, **(B)** VS56, **(C)** VS10, **(D)** VS12, **(E)** VS18, and **(F)** VS16. The serratiopeptidase, standard chromatogram has a retention time of 4.56 min. The HPLC chromatogram of VS56 showed a peak at a retention time of 4.66 min.

The presence of serratiopeptidase in the crude cell-free supernatant was confirmed by HPLC analysis. The retention time under similar conditions of both standard and samples was scrutinized to identify the presence of serratiopeptidase. The HPLC chromatogram of VS56 showed a peak at a retention time of 4.51 min, which is exactly the same as standard having a peak at 4.58 min. The retention time of VS10 was found to be 4.71 min, whereas VS12 and VS18 had a retention time of 4.66 and 4.59 min, respectively (Figure 5). The strain VS16 showed a closer retention time of 4.67 min when compared with standard. This confirmed the presence of serratiopeptidase in the crude sample of VS10, VS12, VS18, VS16, and VS56.

### 3.5 Anti-inflammatory activity

**Figure 6:**
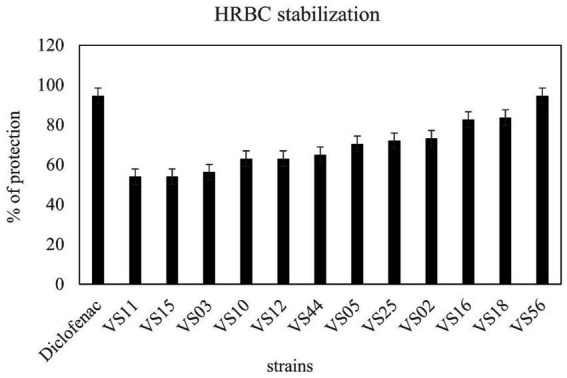
Anti-inflammatory activity of isolates from various sources and diclofenac as control.

A total of 12 strains were examined further for anti-inflammatory activity. The anti-inflammatory activity of strains was considered a remarkable ability in the selection of strains. HRBC stabilization assay was adopted to evaluate the extent of anti-inflammatory activity. The results showed that waste soil isolate VS56 had a maximum percentage of RBC protection (94.6 ± 1.01; Figure 6). The percentage of RBC protection was equivalent to the standard diclofenac (94.6% ± 1.01). The strains VS16 (83.60 ± 1.00) and VS18 (82.70 ± 1.01) also had a significant percentage of anti-inflammatory activity. While VS02 (73.20 ± 1.01), VS25 (72 ± 1.00), VS05 (70.40 ± 1.01), VS02 (73.2 ± 1.00), VS05 (70.4 ± 1.01), and VS25(72 ± 1.10) showed a moderate amount of ant-inflammatory activity. VS44 (65 ± 1.00), VS12(63 ± 1.01), and VS10 (63 ± 1.01) had moderate anti-inflammatory activity, whereas the other strains VS03, VS11, and VS15 exhibited least anti-inflammatory effect. The other selected protease-positive strains, VS16 and VS18 had 83% RBC protection, indicating optimum anti-inflammatory activity. Out of all the protease-positive strains, VS15 had the least (54 ± 1.01) anti-inflammatory activity.

### 3.6 Clot lysis activity

**Figure 7:**
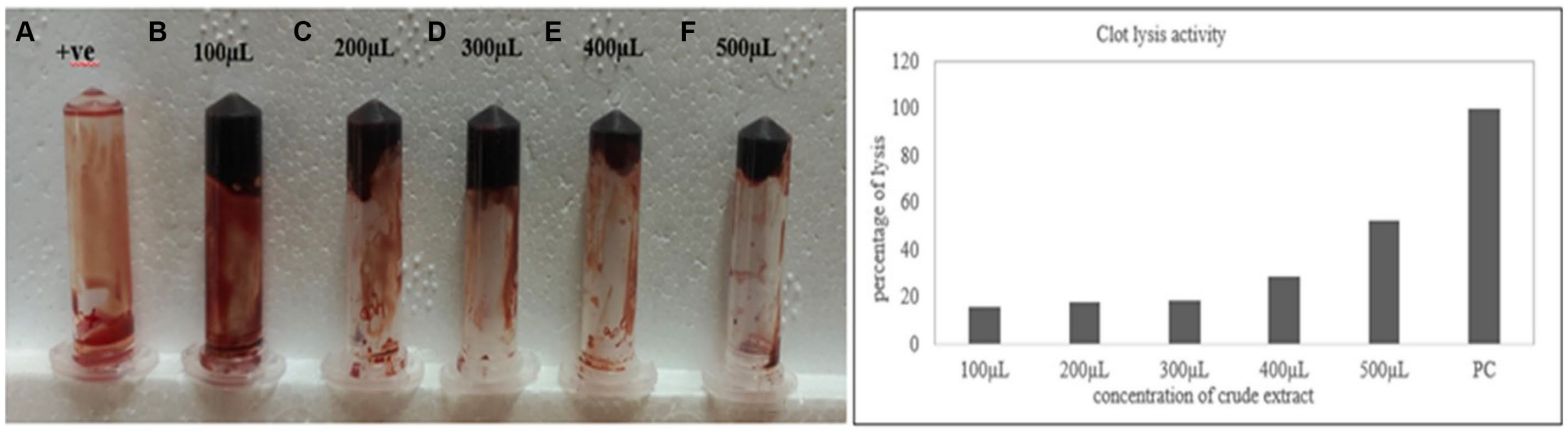
**(A)** Clot lysis of VS56. **(B)** streptokinase c (PC-positive control) with 100% lysis after 30 min **(B)** 100 μL, **(C)** 200 μL, **(D)** 300 μL, **(E)** 400 μL, and 500 μL **(f)** of supernatant.

The clot lysis percentage of *S. marcescens* VS56 was observed to be 53% (Figure 7). In comparison with different volumes of cell-free suspension of a 24 h grown culture, maximum clot lysis was observed in 500 μL. The positive control streptokinase C (100 μL), had 100% lysis within 30 min. The selected potent strain,

*S. marcescens* VS56, thus has moderate clot lysis activity.

### 3.7 SDS-page

**Figure 8:**
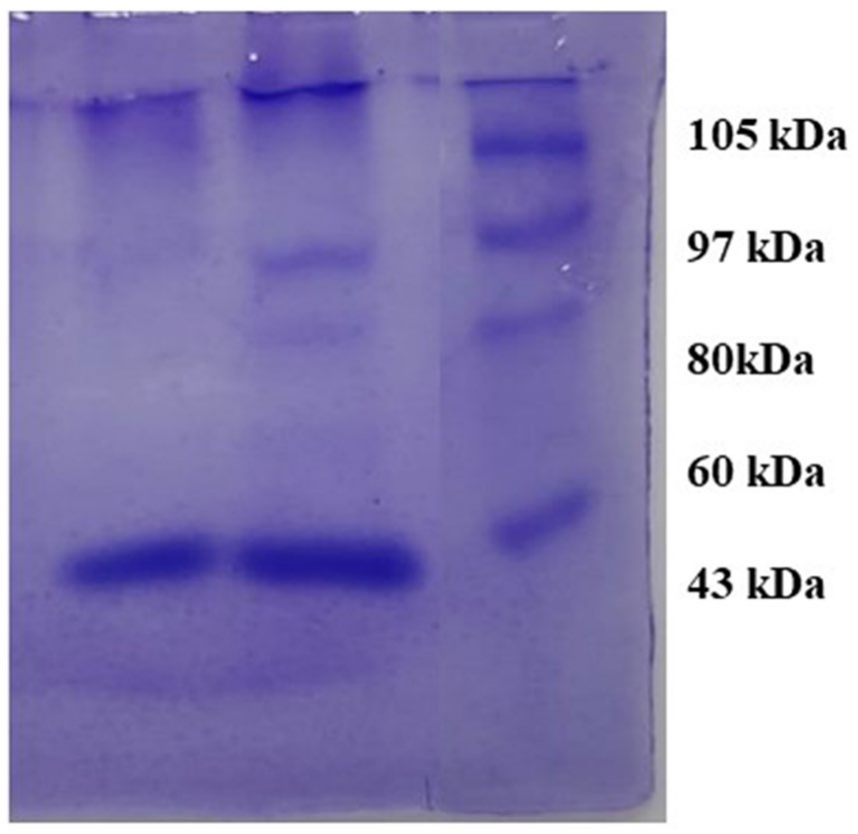
SDS PAGE of VS56 showing protein band between 50 and 70kb indicating the presence of serratiopeptidase Lane1 [L1]: Marker protein, Lane 2 [L2]: crude enzyme and Lane 3 [L3]: Purified enzyme.

The observed bands in crude and purified samples of *S. marcescens* VS56 are identical to 60kDA in the protein marker. The molecular weight of the protein produced by *S. marcescens* VS56 was identified to be approximately 60 kDA (Figure 8), which indicates the presence of serratiopeptidase. The band observed at the sample lane hence confirmed the presence of serratiopeptidase production by

*S. marcescens* VS56 (Figure 8).

## 4 Discussion

Serratiopeptidase is a potent serine protease usually isolated from sp. *Serratia*. Considering the pharmaceutical importance and industrial demand of this enzyme, the present study has focused on the identification of a potent serratiopeptidase-producing strain. There are many existing reports about different organisms producing serratiopeptidase (Fritsch et al., 2013). It includes a recombinant *E. coli* BL21 (DE3) as well as the wild strain *S. marcescens* e-15 (Anil and Kashinath, 2013). *S. marcescens* VS56 in the study was isolated from untreated waste soil and also observed as a potent producer of serratiopeptidase. According to Rebecca et al. (2013), soil serves as a rich source for isolating *Serratia* sp. they have successfully isolated *S. marcescens* from soil sources and screened for chitinase activity. According to Muthukumar et al. (2016), soil sources are regarded as safe compared to wounds or infection sites. As *S. marcescens* VS56 of this study is from soil, it can be regarded as safe to handle. This increases the safety of handling this bacterium for the industrial production of serratiopeptidase. Anil and Kashinath (2013) estimated the protein content and protease activity of *S. marcescens* e15 and observed 0.083 mg/mL of secretory proteins in it. *S. marcescens* VS56 has a total protein content of 102 mg/mL. The strain has more secretory protein in the cell-free supernatant. Enzymatic activity was observed using casein as a substrate (Salisbury and Likos, 1972), where *S. marcescens* VS56 has 12.31 units/mL of active enzyme. When compared with the study of Koul et al., 2021 the isolates *S. marcescens* MES-4 and *S. marcescens* MRS-11 from mulberry soil had protease activity of 95 units/mL and 156 units/mL, respectively. Currently, wild or mutant strains of *S. marcescens* are used to meet the industrial and pharmaceutical need for serratiopeptidase. Rebecca et al. (2013), in their study, have mentioned the significance of source selection for the isolation of *Serratia*. According to Rebecca et al. (2013), fish market soil served as a potent source for isolating *Serratia*. In the present study, the strains *C. amylolyticum* VS10 and *P. psychrophila* VS16 were isolated from fish and butcher house soil, respectively. While the other serine protease-positive strains, *K. rosea* VS12 *and K. rosea* VS18, were isolated from coconut waste and sugarcane. The cell-free supernatant contains all the secretory proteins, and its activity was estimated by protease activity and anti-inflammatory activity. The anti-inflammatory potential of bioactive compounds has been explored by Muthukumar et al. (2016), and there was 88% RBC protection. The anti-inflammatory potential of crude *S. marcescens* VS56 was 94.6% equivalent to the standard diclofenac (94.6%). The isolate not only has protease activity but a strong anti-inflammatory activity too. HPLC has been regarded as the most explored analytical tool used in the identification of serratiopeptidase. Navneet Kumar et al. (2017) study used the standard serratiopeptidase, which showed a retention time of 5.94 min. These data validate the ease and specificity of comparing the presence of serratiopeptidase in the crude sample. A similar approach was used in this study, where all the cell-free crude was examined for the presence of serratiopeptidase by comparing the retention time. The standard serratiopeptidase showed a retention time of 4.59 min. Compared with this, *S. marcescens* VS56 showed a major peak at 4.51 min. VS10, VS12, and VS18 also have major peaks at 4.04, 4.51, and 4.71 min, respectively. This could be effective proof to confirm the presence of serratiopeptidase in the crude extract of these selected isolates. Krishnamurthy and Belur (2018) studied the clot lysis potential of *S. marcescens* subsp. *sakuensis* and found 37% of clot lysis potential. The incubation condition and time for the study were 37°C for 4 h. Compared to this, *S. marcescens* VS56 was able to lyse 53% of the clot after 4 h. This marked the fibrinolytic potential of *S. marcescens* VS56. Serratiopeptidase is a large protease enzyme with a molecular weight of 50–60 kDa. Chander et al. (2021) have specifically identified that serratiopeptidase possesses a molecular weight of 51 kDa (Braun and Schmitz, 1980). When compared with this study, the potent soil isolate *S. marcescens* VS56 showed a band between 50 and 60 kDa, which indicates the presence of serratiopeptidase. The soil isolate *S. marcescens* VS56 can be used as an alternative potent bacterium for mass industrial production.

## 5 Conclusion

The study was to explore the serratiopeptidase-producing strain in various samples. Several bacterial isolates from different samples were screened for serratiopeptidase production. The serratiopeptidase-producing ability of the different bacterial isolates was compared. Based on the research findings, *S. marcescens* VS56 from soil has been identified as a potent serratiopeptidase producer. In the future, this novel strain could be beneficial to bacterial culture in the pharmaceutical industry to achieve enhanced production of serratiopeptidase.

## Data availability statement

The original contributions presented in the study are included in the article/Supplementary material, further inquiries can be directed to the corresponding author.

## Author contributions

SN: Validation, Data curation, Formal analysis, Investigation, Writing – original draft. CS: Validation, Conceptualization, Funding acquisition, Supervision, Writing – review & editing.
