# Supplementary material for: Bioprospecting of serratiopeptidase-producing bacteria from different sources
Source: Front Microbiol. 2024 May 9;15:1382816. doi: 10.3389/fmicb.2024.1382816 (PMC11123226; doi:10.3389/fmicb.2024.1382816)
Supplement: Supplementary file 1 [file Table_1.DOCX]

**SUPPLEMENTARY DATA**

**BIOPROSPECTING OF SERRATIOPEPTIDASE PRODUCING BACTERIA FROM DIFFERENT SOURCES**

Sreelakshmi R Nair^1^, Subathra Devi C^2*^

^1^Department of Biotechnology, School of Bio Sciences and Technology, Vellore Institute of Technology, Vellore, Tamil Nadu, India.

*** Corresponding author:** Subathra Devi. C

[subaresearch@rediffmail.com](mailto:subaresearch@rediffmail.com)


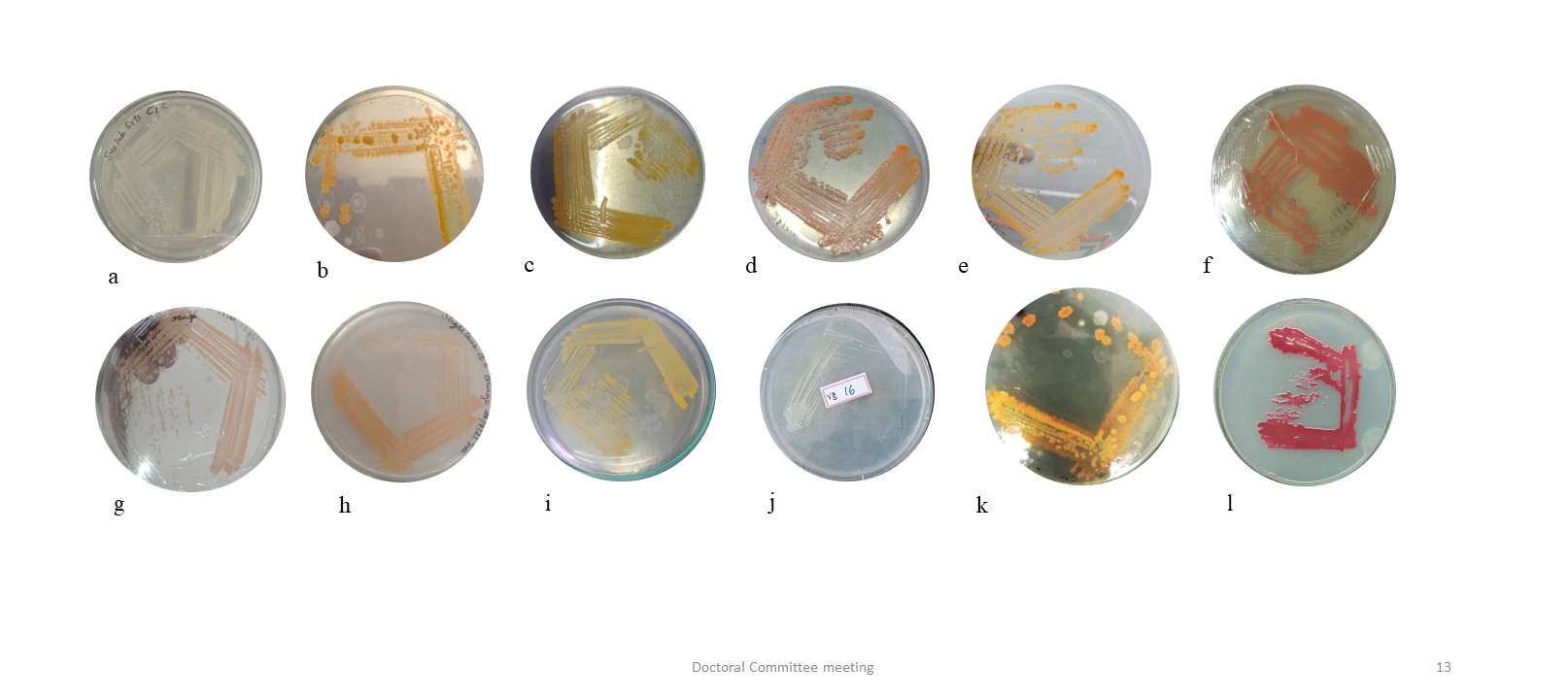


Fig. S1. Pure culture of a) VS02, b) VS03, c) VS05, d) VS10, e) VS11, f) VS12, g) VS15, h) VS16, i)VS18, j)VS25, k)VS44 and l)VS56 on nutrient agar medium.


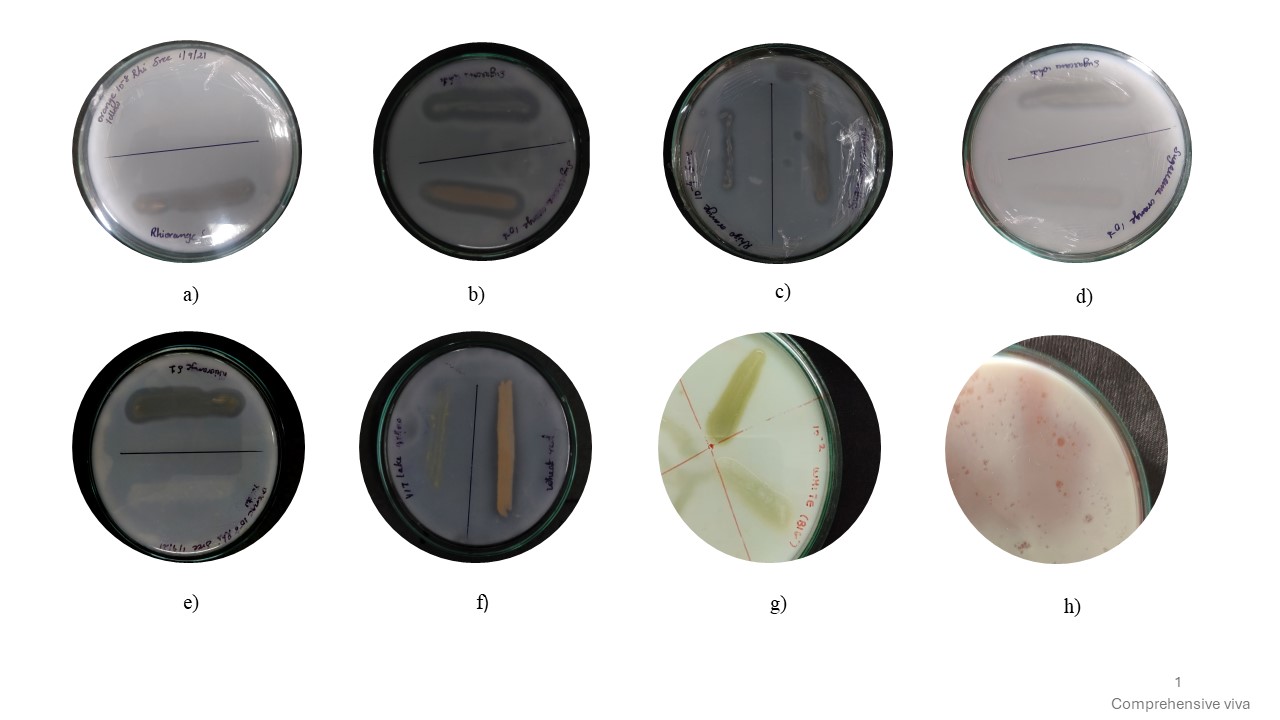


Fig.S2. Protease activity of a) VS02, b) VS03, c) VS05, d) VS10, e) VS11, f) VS12, g) VS15, h)VS16, i)VS18, j)VS25, k)VS44 and l)VS56 on skim milk agar
